# Hypothenar Lipoma Presenting With Ulnar Neuropathy: A Rare Case and Review of Surgical Management

**DOI:** 10.7759/cureus.92725

**Published:** 2025-09-19

**Authors:** Mohamed Elsakka, Nayef Louri

**Affiliations:** 1 Plastic and Reconstructive Surgery and Burns Unit, Bahrain Defence Force Royal Medical Services Military Hospital, Riffa, BHR

**Keywords:** hand tumors, hypothenar lipoma, soft tissue neoplasm, surgical excision, ulnar nerve compression

## Abstract

Lipomas of the hand are rare, particularly in the hypothenar region. We present a case of a 43-year-old male patient with a slow-growing, painless mass in the right hypothenar area causing intermittent tingling and paresthesia in the ulnar nerve distribution. Magnetic resonance imaging (MRI) confirmed a lipomatous lesion. Surgical excision revealed the mass adherent to the superficial branch of the ulnar nerve. Histopathology confirmed a benign lipoma. The patient experienced complete sensory recovery six months postoperatively, with no recurrence. This report discusses the importance of early diagnosis and nerve-sparing excision in hypothenar lipomas.

## Introduction

Lipomas are the most common benign soft-tissue tumors, accounting for nearly 50% of all soft-tissue neoplasms. However, their occurrence in the hand, especially the hypothenar region, is uncommon, representing only 1% to 3.8% of tumors in this anatomical area [1].

When present, they can remain asymptomatic or produce compressive symptoms depending on their size and anatomical relationships. Compression of the ulnar nerve by a hypothenar lipoma is particularly rare but clinically significant because of its functional implications on hand sensation and dexterity [2-5]. This case highlights a rare presentation and reviews imaging, surgical management, and recovery.

## Case presentation

A 43-year-old right-handed man presented with a one-year history of a painless, progressively enlarging mass in the right hypothenar area, associated with intermittent numbness and tingling in the ring and little fingers. There was no trauma, infection, or prior surgical history.

**Figure 1:**
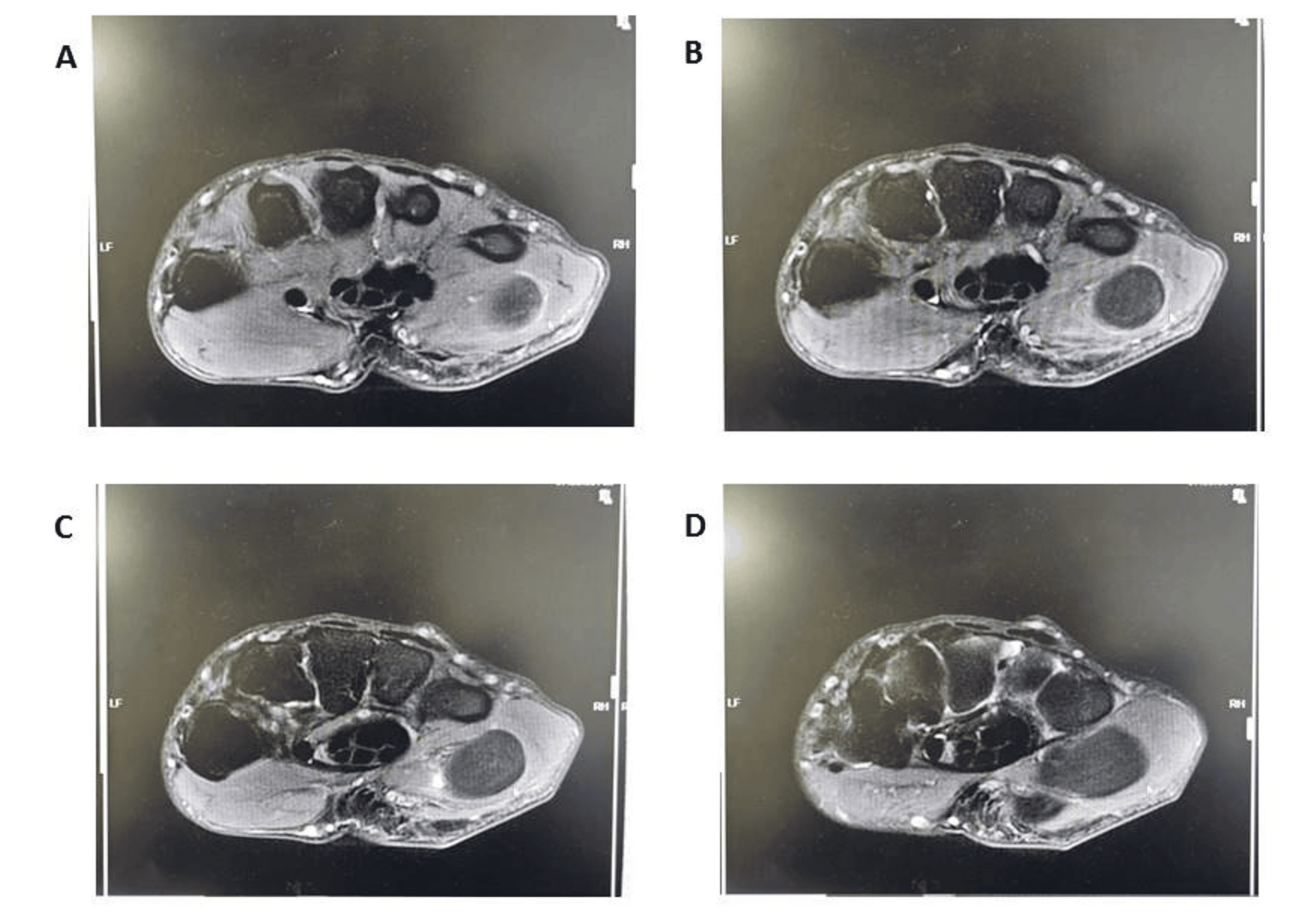
Sequential axial MRI images of the hypothenar region demonstrating a well-circumscribed, hyperintense soft tissue mass on T1-weighted sequences, consistent with lipoma

**Figure 2:**
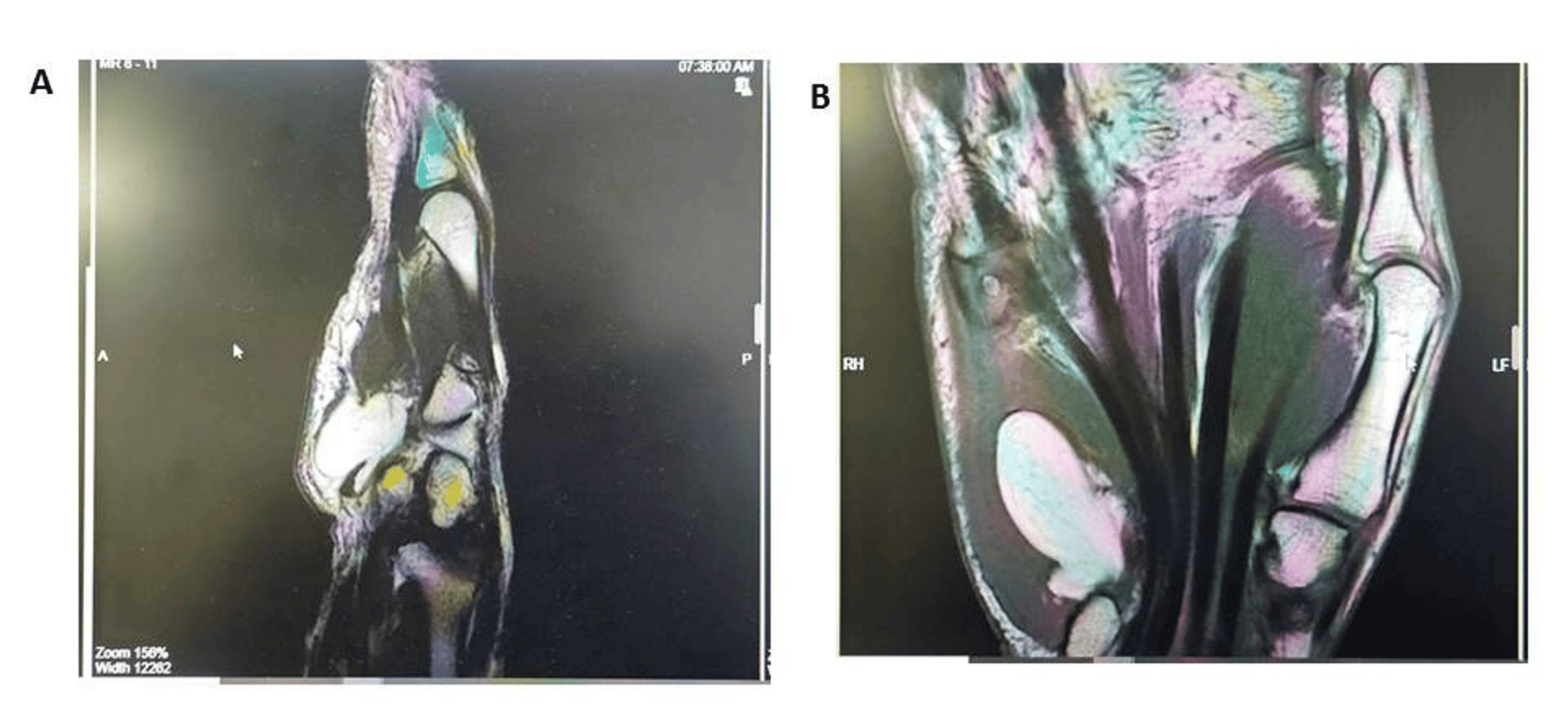
Coronal and sagittal planes of MRI findings of lipomatous mass

Examination revealed a soft, mobile, non-tender mass measuring approximately 4 × 5 × 1.5 cm in the right hypothenar region. Neurological assessment demonstrated mild sensory disturbance in the ulnar nerve distribution. Motor examination showed normal bulk and contour of the thenar, hypothenar, and intrinsic hand muscles, with no evidence of wasting or fasciculations. Muscle strength was preserved, graded as 5/5 in all ulnar-innervated intrinsic muscles. MRI revealed a well-encapsulated, homogeneous high-signal lesion on T1 and T2-weighted images, consistent with a lipoma compressing the superficial branch of the ulnar nerve (Figures 1, 2).

Surgical management

**Figure 3:**
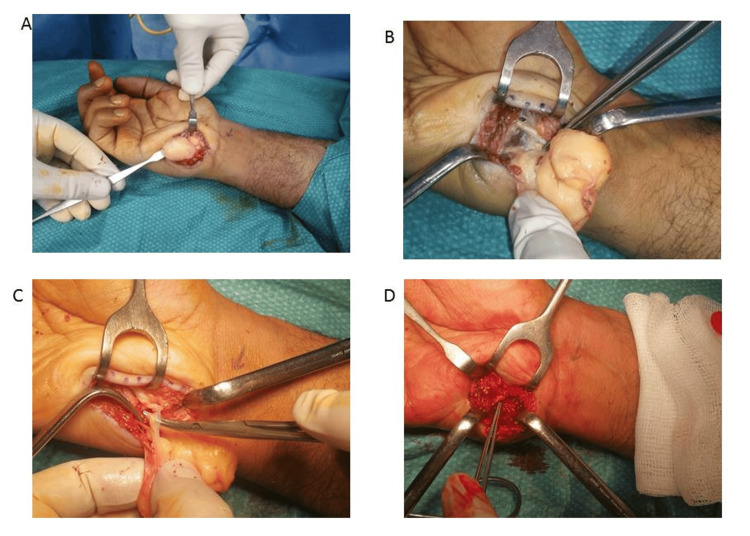
Operative dissection of the lipomatous mass A: Intraoperative identification of the lipomatous mass; B: Dissection around the lipomatous mass; C: Relation of the lipomatous mass to the surrounding nerve structure; D: Operative field post complete mass excision

**Figure 4:**
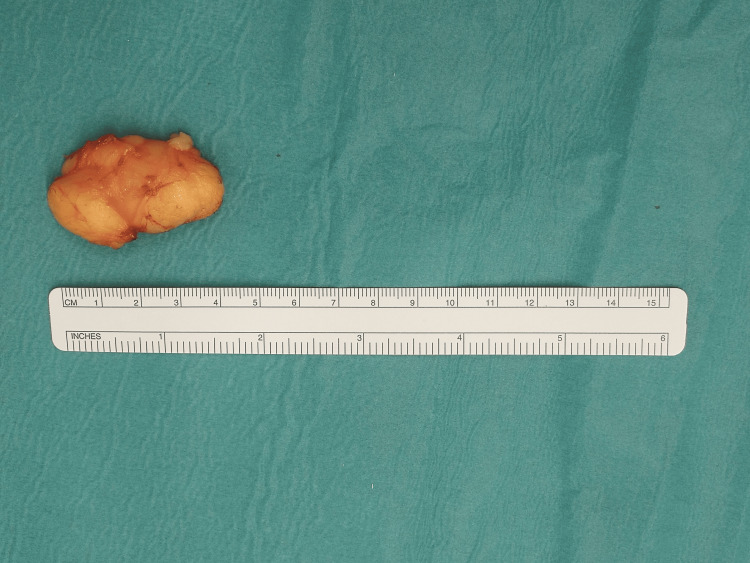
Dimension of the excised lipomatous mass

Under general anesthesia, the lesion was carefully excised via a volar ulnar approach, employing tourniquet control to maintain a bloodless field and loupe magnification to enhance visualization of delicate neurovascular structures. Intraoperatively, a well-encapsulated lipoma was identified adjacent to and partially adherent to the superficial branch of the ulnar nerve. Careful microsurgical dissection was performed to separate the mass from the nerve without causing neural injury. The surgical exposure did not extend into Guyon’s canal, and no exploration of the canal was undertaken. This decision was based on the absence of clinical and radiological findings of ulnar tunnel involvement, the preservation of normal motor function (5/5 strength in all ulnar-innervated intrinsic muscles), and MRI findings that confirmed the lesion was confined to the hypothenar region without extension into the canal. Careful microdissection preserved the nerve. The mass was excised en bloc. Histopathology confirmed a benign mature lipoma (Figures 3, 4).

Postoperative outcome

**Figure 5:**
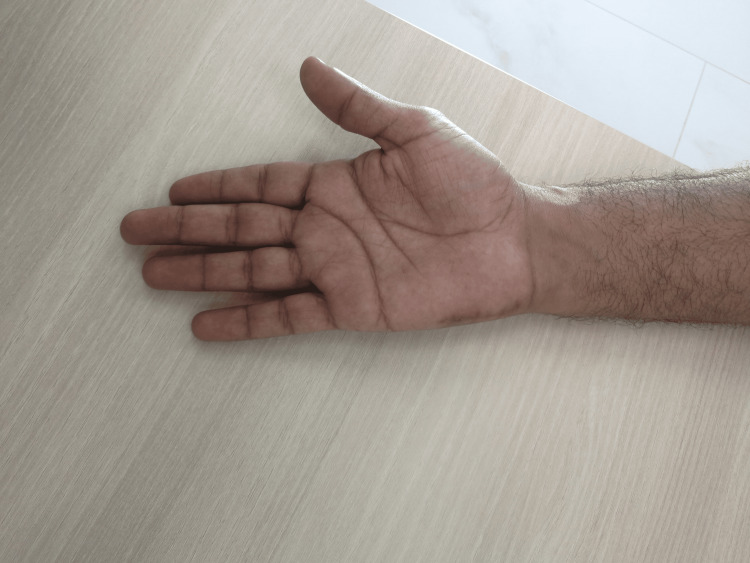
Postoperative scar at six months

The patient recovered uneventfully. Sensory symptoms improved over several weeks, with full resolution at six months. No recurrence was observed, and the scar healed with excellent cosmesis (Figure 5).

## Discussion

Lipomas of the hypothenar region are rare and should be considered in the diagnostic work-up of soft tissue masses in this area, particularly when associated with unexplained ulnar nerve symptoms. The differential diagnosis includes both benign and malignant entities, such as liposarcoma, hamartoma, angiolipoma, and hibernoma, necessitating careful clinical, radiological, and histopathological evaluation to ensure accurate diagnosis and appropriate management. Although lipomas are common benign soft tissue tumors, their occurrence in the hand and particularly in the hypothenar area is unusual. This rarity can delay diagnosis, especially when the mass is deep-seated and only becomes symptomatic once it causes compression of nearby neurovascular structures [6].

Consistent with these observations from the literature, our patient presented with intermittent ulnar nerve-related symptoms, particularly paresthesia in the ring and little fingers. The lesion’s deep-seated position within the hypothenar region contributed to the delay in recognition, as no obvious surface mass was initially detected. Compared with thenar lipomas, hypothenar lesions are encountered far less frequently, and their close relationship to vital structures such as the ulnar nerve and Guyon’s canal makes both diagnosis and surgical management more challenging.

Electrodiagnostic testing nerve conduction studies, combined with needle electromyography, are a valuable adjunct in the evaluation of suspected peripheral mononeuropathies because it can confirm the presence of ulnar neuropathy, localize the site of involvement, quantify severity, and distinguish focal entrapment from diffuse neuropathic processes. In cases with unequivocal clinical and radiological evidence of a localized, superficial lesion confined to the hypothenar region and preserved motor strength (5/5 in ulnar-innervated muscles), routine preoperative electrodiagnostic testing is not universally indicated. However, it may be considered for lesion localization (including Guyon’s canal involvement), establishing a preoperative baseline, or when clinical findings are subtle or progressive [7,8].

MRI played a vital role in this case by clearly identifying the lesion's encapsulated nature, its homogeneity on T1- and T2-weighted images, and its anatomical relationship with the superficial branch of the ulnar nerve. MRI is considered the imaging modality of choice for soft tissue tumors of the hand due to its ability to delineate tissue planes and neurovascular relationships without the need for ionizing radiation [9]. Although ultrasound is a cost-effective and readily accessible tool for initial evaluation of superficial soft tissue masses, it is highly operator-dependent and has limited ability to assess deep lesions or provide comprehensive anatomical details. Given the deep location of the hypothenar mass and the clinical suspicion of nerve involvement, MRI was favored over ultrasound to ensure accurate diagnosis and optimal preoperative planning.

Surgical management must prioritize meticulous dissection and nerve preservation, especially when the mass is adherent to or closely associated with a nerve. In this case, although the lipoma was partially adherent to the superficial branch of the ulnar nerve, careful microdissection allowed for en bloc excision without damaging the nerve. This approach differs from the management of nerve sheath tumors, which may require different operative considerations.

The findings in this case align with similar reports in the literature. For instance, Sreekumar et al. (2014) described a lipoma causing Guyon’s canal syndrome [2], while Kim et al. (2019) reported a giant intramuscular lipoma in the hand [3]. However, reports that specifically detail isolated hypothenar lipomas with ulnar involvement and favorable nerve-sparing outcomes remain scarce. This case contributes to the growing body of evidence supporting early surgical intervention and nerve-sparing techniques in such scenarios.

Furthermore, intramuscular lipomas of the hypothenar eminence are exceedingly rare, with only a few cases reported. Lee et al. (2004) described two hypothenar intramuscular lipomas in a series of six hand lipomas, highlighting their deep-seated nature, average size of approximately 5.5 cm, association with grip weakness, and successful excision under loupe magnification without recurrence [10]. Similarly, de Magalhães et al. (2020) reported a well-encapsulated intermuscular hypothenar lipoma causing mild ulnar nerve compression, which was effectively managed with surgical resection and resulted in complete resolution of symptoms [11]. A more recent case by Kumar et al. (2024) described a giant intramuscular lipoma in the hypothenar region presenting with tingling and numbness in multiple fingers, which was excised while preserving the superficial ulnar nerve, resulting in full recovery [12]. These reports reinforce the rarity of hypothenar lipomas, their potential for neurological involvement, and the importance of careful preoperative imaging and meticulous surgical dissection. The present case aligns with these findings, demonstrating that early recognition and nerve-sparing excision can achieve excellent functional outcomes while preventing long-term neuropathic complications.

## Conclusions

This case highlights the importance of considering lipoma in the differential diagnosis of hypothenar masses with neurological symptoms. MRI is essential for diagnosis and surgical planning. Prompt surgical excision with nerve preservation can yield excellent functional outcomes.
